# Supplementary material for: Changes in soil phosphorus dynamics amended with rock phosphate-enriched compost and chemical fertilizers
Source: PLoS One. 2025 Jun 3;20(6):e0324716. doi: 10.1371/journal.pone.0324716 (PMC12133002; doi:10.1371/journal.pone.0324716)
Supplement: Attachment — Submitted filename: Comments for the Author.docx [file pone.0324716.s001.docx]

**Comments for Authors:**

This study explored the impact of phosphorus management strategies on mustard production. It found that the combination of 100% recommended fertilizer dose (RDF) with enriched compost significantly improved phosphorus availability and soil microbial activity, leading to a 16.7% higher mustard grain yield compared to control. The results suggest that this combination enhances P accumulation and soil-plant P transformations, benefiting yields in degraded soils. Enriched compost at 6 t ha-1 also improved microbial biomass and enzymatic activity, supporting sustainable agricultural practices. The work is a meaningful contribution to agricultural modeling and is suitable for publication with minor revisions.

**Specific comments:**

1. The title is very suitable for the study, no need to change the title.

2. The abstract of the study is sufficient.
3. Line 6, the abbreviation should be elaborated when using first time.
4. Line 8, change the “we” and check the grammar.
5. Please arrange the Keywords in alphabetical order.
6. The introduction is inappropriate. The first paragraph is overly long, while the others are too short. Please review and reorganize the introduction section. Adjust the language to ensure better clarity and coherence. Also inroduce Mastard juncea in few lines.
7. Line number 108, Reference is not according to journal format.
8. The study shows that combining enriched compost (6 t ha⁻¹) with 100% RDF boosts Olsen-P, total P, inorganic P, and microbial activity compared to the control. However, enriched compost alone cannot fully replace water-soluble P fertilizers. Could the authors explain the limitations of enriched compost in replacing chemical P fertilizers? Also, what are the long-term effects of using enriched compost for P availability in soils with low P content or high P fixation?
9. There is a problem with using abbreviations. The full term should be mentioned first with the abbreviation between paresis then the abbreviations should be exclusively used throughout the manuscript. Please revise the whole paper’s abbreviations carefully and correct them accordingly.
10. I reviewed the layout of the article: I would suggest the tables and figures should appear where they are mentioned in the text the first time (in Results) and not several pages later.
11. Please use the same and uniform text size of heading and footnotes for all Tables and Figures throughout the paper.
12. Please avoid symbols at the beginning of the sentences.
13. Revise the conclusion and summarize it according to the key findings of your study.
14. Please arrange all the references according to the Journal format.
